# Investigating Mechanical Behaviours of PDMS Films under Cyclic Loading

**DOI:** 10.3390/polym14122373

**Published:** 2022-06-12

**Authors:** Kyu Song, Nak-Kyun Cho, Keun Park, Chung-Soo Kim

**Affiliations:** 1 Department of Manufacturing Systems and Design Engineering, Seoul National University of Science and Technology (SeoulTech), Seoul 01811, Korea; songkyu@seoultech.ac.kr; 2 Department of Mechanical Systems Design Engineering, Seoul National University of Science and Technology (SeoulTech), Seoul 01811, Korea; kpark@seoultech.ac.kr; 3 Advanced Forming Process R&D Group and 3D Printing Manufacturing Process Centre, Korea Institute of Industrial Technology (KITECH), Ulsan 31056, Korea; cskim91@kitech.re.kr

**Keywords:** polydimethylsiloxane (PDMS) film, cyclic mechanical property, cyclic tensile behaviour, strain-controlled cyclic test, hyperelastic material coefficient

## Abstract

Polydimethylsiloxane (PDMS) is widely utilised as a substrate for wearable (stretchable) electronics where high fatigue resistance is required. Cyclic loadings cause the rearrangement of the basic molecular structure of polymer chains, which leads to changes in the mechanical properties of the PDMS structure. Accordingly, it is necessary to investigate reliable mechanical properties of PDMS considering both monotonic and cyclic loading conditions. This study aims to present the mechanical properties of PDMS films against both monotonic and cyclic loading. The effects of certain parameters, such as film thickness and magnitude of tensile strain, on mechanical properties are also investigated. The test results show that PDMS films have a constant monotonic elastic modulus regardless of the influence of thickness and tensile loading, whereas a cyclic elastic modulus changes depending on experimental parameters. Several material parameters, such as neo-Hookean, Mooney–Rivlin, the third-order Ogden model, and Yeoh, are defined to mimic the stress–strain behaviours of the PDMS films. Among them, it is confirmed that the third-order Ogden model is best suited for simulating the PDMS films over the entire tensile test range. This research makes contributions not only to understanding the mechanical behaviour of the PDMS films between the monotonic and the cycle loadings, but also through providing trustworthy hyperelastic material coefficients that enable the evaluation of the structural integrity of the PDMS films using the finite element technique.

## 1. Introduction

Polydimethylsiloxane (PDMS) is a transparent, hydrophobic and isotropic material [1]. Because of its advantages, such as high elongation, high fatigue endurance and environmental and human friendliness [2,3,4], PDMS has been used in a wide range of industries such as electronics, machinery and biomedical engineering [5]. Film-type PDMS has been used as a substrate in products of wearable electronics and flexible electronics [6,7]. In another form, PDMS composites with carbon nanotubes (CNT-PDMS) have been widely used in the manufacturing of stretchable sensors [8]. Increasing demand for PDMS in many industries is driving the growth of the PDMS market, which is estimated to reach USD 5.2 billion by 2024.

Mechanical properties of PDMS vary depending on various manufacturing conditions, such as curing temperature, curing time and curing agent ratio [9,10,11]. Additionally, the structural performance of PDMS products is commonly assessed using actual tests with a real size product [12]. Although the real tests can predict reliable mechanical behaviour of PDMS products, it may be expensive to involve all the test variables. Hence, it is necessary to obtain trustworthy mechanical properties of PDMS and evaluate the structural integrity of PDMS products using simulation techniques.

Generally, PDMS shows nonlinear mechanical behaviours in the elastic region, unlike metallic materials [13]. Many researchers have reported that the mechanical properties of PDMS are affected by the following parameters: manufacturing processes, the thickness of PDMS specimen and the magnitude of applied loading. Liu et al. (2009) studied the effects of PDMS thickness on the elastic modulus and mechanical behaviours under monotonic tensile loadings. In their follow-up study, the effects of PDMS thickness on the mechanical behaviours of PDMS and cyclic softening behaviours under cyclic tensile loading were presented [14]. Johnston et al. (2014) showed interesting findings, that the curing temperature induced a difference in the mechanical properties of PDMS under tension and compression [15]. Zakaria et al. (2017) demonstrated that curing time is an important factor that affects mechanical properties of several elastomers, including PDMS [16]. Muller et al. (2019) introduced a method for determining Poisson’s ratio and the coefficient of thermal expansion (CTE) for two types of PDMS (Sylgard 182 and 184) using an optical surface profilometer [17]. Bedon and Mattei (2021) investigated the advantages of operational modal analysis (OMA) techniques by obtaining the elastic modulus of multilayer anti-shatter safety films (ASFs) [18]. Mattei et al. (2022) analysed the effects of strain rate and specimen aging on the mechanical properties of ASFs based on ASTM-D882 [19]. However, the aforementioned studies only considered monotonic loading, without considering cyclic loading, to derive the mechanical properties of the PDMS, or suggested only the difference in the mechanical characteristics through mutual comparison between specimens against experimental variables. Therefore, it is necessary to define the mechanical properties of PDMS films under cyclic loading conditions.

This research aimed to obtain the mechanical properties of PDMS films with thicknesses of 150 μm, 200 μm and 250 μm, which were subjected to cyclic tensile loading. Based on the tensile test results, differences in elastic modulus between monotonic loading and cyclic loading were quantitatively defined. Comprehensive parametric studies were conducted to demonstrate the effects of film thickness and magnitude of load displacement on the elastic modulus and peak stress. Moreover, material coefficients were derived against each test variable to simulate hyperelastic behaviours of the PDMS films using CAE software Abaqus ver.2020.

This paper is structured as follows: Section 2 introduces the PDMS materials and the cyclic tensile test method as well as the manufacturing process of the PDMS film and the experimental conditions are described. Section 3 presents the mechanical properties of each PDMS film and compares the properties between monotonic loading and cyclic loading. Section 4 includes the material coefficients that enabled us to simulate the obtained hyperelastic behaviour of each PDMS film. Section 5 presents the concluding remarks of this research.

## 2. Materials and Testing Methods

**Figure 1:**
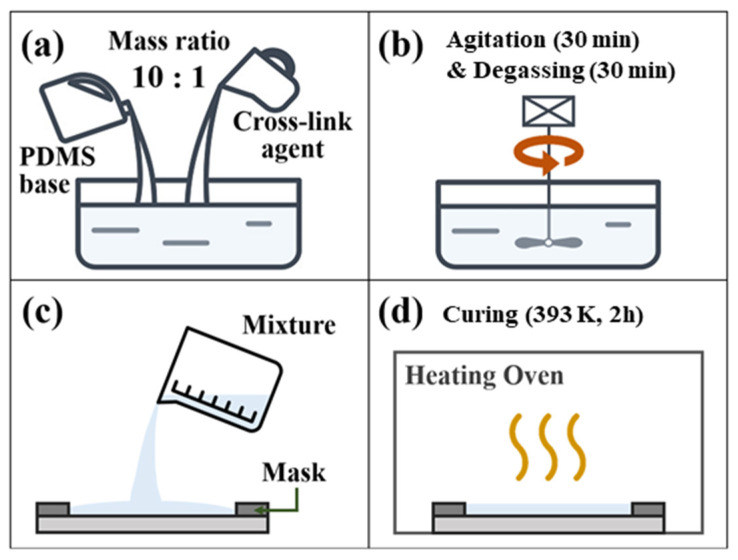
Schematic diagram of the manufacturing process: (**a**) PDMS mixing, (**b**) agitation & degassing process, (**c**) pouring PDMS mixture into a cavity and (**d**) curing process.

All testing specimens used in this study were fabricated using Sylgard 184 (Dow Corning Corp., Midland, MI, USA). Figure 1 illustrates the manufacturing process of the specimens. The mass ratio of PDMS to the cross-link agent was 10:1 and we mixed the two materials for 30 min using an electric agitator. The deforming process, that removes air bubbles from inside the mixture, was conducted for 30 min in a vacuum environment. The PDMS mixture was poured in a designed cavity made by a mask with different thicknesses, as shown in Figure 1c. The specimen was cured for 2 h in a heating oven with a constant temperature maintained at 393 K to finish the preparation of the specimens.

**Figure 2:**
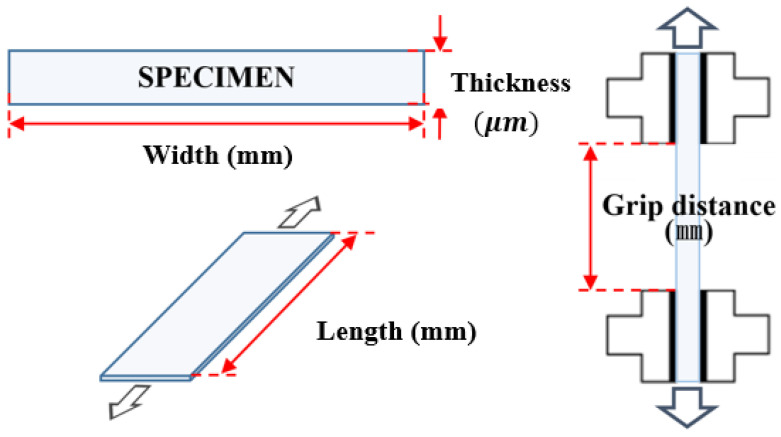
Schematic diagram of the specimen dimensions.

Figure 2 depicts the key geometric parameters of the specimens and the parameters are listed in Table 1. To evaluate the effects of the film thickness on the cyclic tensile behaviour, the width and the length were set to fixed dimensions. The specimens with thicknesses of 150 μm, 200 μm and 250 μm, within a thickness tolerance of ± 10 μm, were fabricated using the manufacturing process shown in Figure 1 and were employed for the cyclic tensile test. The cyclic tensile experiment was conducted in accordance with ASTM-D882 (standard test method for tensile properties of thin plastic sheeting), in which the grip distance and the length of the specimen were set to 50 mm and 100 mm, respectively [20].

**Figure 3:**
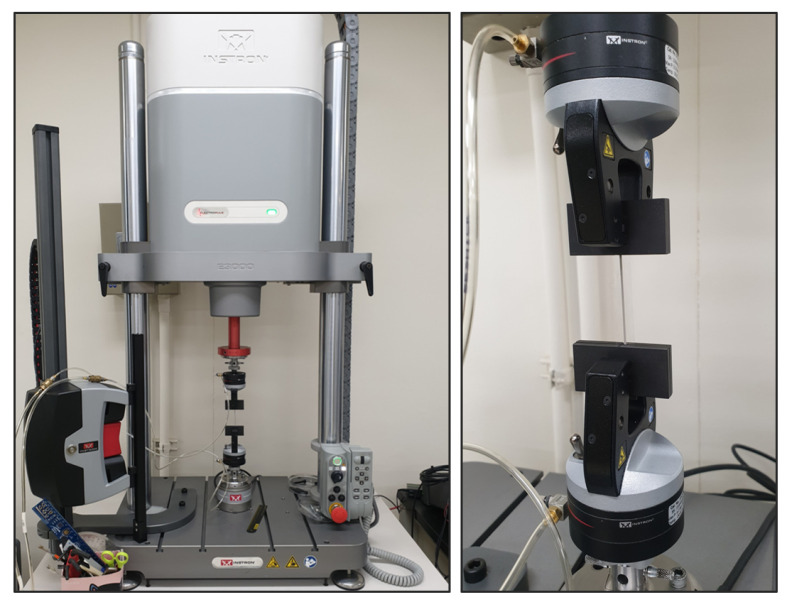
Cyclic tensile test of PDMS films.

Strain-controlled tensile tests were performed, and each specimen was subjected to 20 cycles tensile loadings, during which the experiment defined the initial and cyclic mechanical properties, respectively. The amount of strain in the strain-controlled test was measured using an extensometer included in the test equipment, as shown in Figure 3. During the test, the pneumatic grips constantly maintained a grip strength and prevented between the specimen and the grip. A previous study on a health monitoring system using a stretchable sensor reported that the largest strain that can occur in the human body is 30% [21]. In the case of a polymer film product, the structural performance should satisfy the criteria for high-cycle fatigue endurance [22]. On the other hand, tensile tests for strain of 100% or more were conducted in the development of a stretchable sensor applied to large deformations [23].

In this study, considering future applications of PDMS films, tensile strain ranges were set to from 30% to 130% at a strain rate of 10 mm/min. Based on ASTM-D882, the cyclic tensile test was repeated 5 times to obtain reliable data. Elastic modulus was defined as the slope of the first linear section of the stress–strain curve derived from the cyclic tensile test. Elastic modulus taken from the first cycle and the last cycle (20th) were set as the monotonic elastic modulus (E_m_) and the cyclic elastic modulus (E_c_).

## 3. Test Results and Discussions

**Figure 4:**
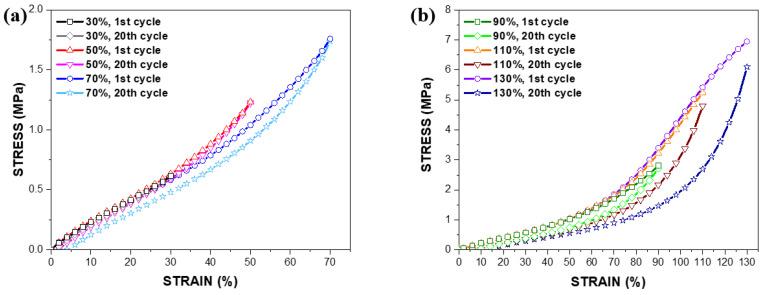
Stress–strain behaviours of the PDMS film with a thickness of 150 μm at the 1st and the 20th cycle against tensile strain loading; (**a**) 30–70% and (**b**) 90–130%.

Figure 4 illustrates the stress–strain behaviours of PDMS films with a thickness of 150 μm at the 1st (first cycle) and the 20th (last cycle) loading cycles. Most stress–strain behaviours of the PDMS film in Figure 4 can be expressed with the third-order polynomial function, whereas the curves at tensile loadings of 110% and 130% require the fourth-order equation to depict their hyperelasticity. Hence, it is understood that an appropriate polynomial function should be employed to simulate the hyperelasticity of the PDMS film with variations in the thickness and a magnitude of the tensile loading.

**Figure 5:**
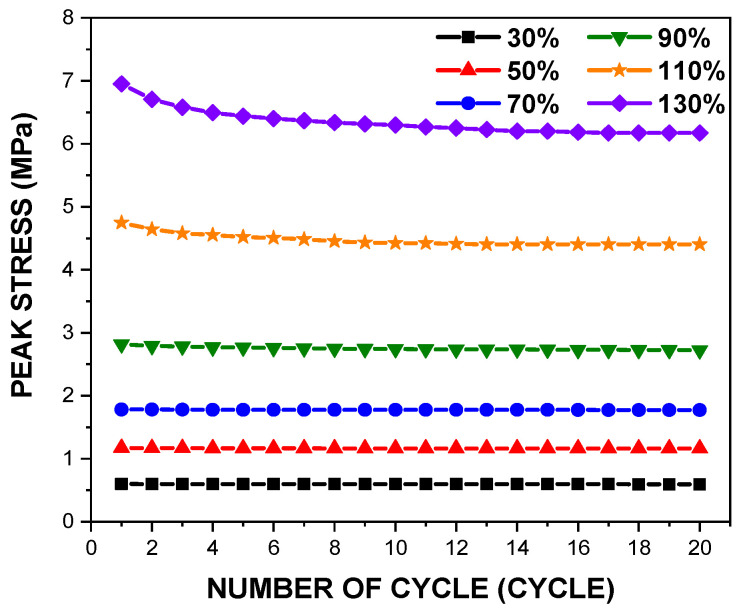
Deviations in the peak stress of the PDMS film with a thickness of 150 μm over the cyclic tensile loading cycles.

Figure 5 depicts variations in the peak stress of PDMS film with a thickness of 150 μm at the 20th loading cycle, at the tensile loading range from 30% to 130%; the results show the stress softening with an increase in the tensile strain loading. The stress softening is a mechanism that occurs when the polymer chain inside the specimen is rearranged by the cyclic tensile behaviour, leading to a change in the stiffness of the specimen [24]. For the range between 30% and 90%, the reduction in the peak stress after the 1st cycle seems very insignificant, but marked decreases were observed for the range between 110% and 130%.

**Figure 6:**
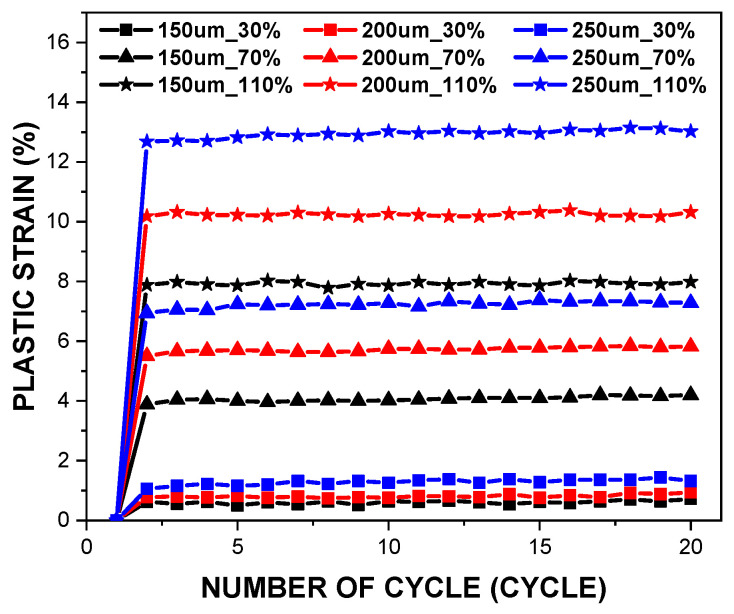
Deviations in plastic strains of all PDMS films subjected to the tensile loading in the elevations.

Figure 6 shows changes in the accumulated plastic strain of all PDMS film specimens over the cyclic tensile loadings. For all specimens, no further significant plastic deformation accumulated over the loading cycles after the initial plastic strain developed at the 1st cycle. In addition, it was found out that the thinner film experienced less plastic deformation than the thicker films at the same tensile loading. Investigation results on the correlation between the stress softening and accumulated plastic strains as the tensile loading increases revealed that they are independent of each other. The increase in the stress softening is an important matter since it can affect the performance of the PDMS film. Hence, it is worth defining the mechanical properties of the PDMS films in the stabilised state against different of tensile loading levels.

**Figure 7:**
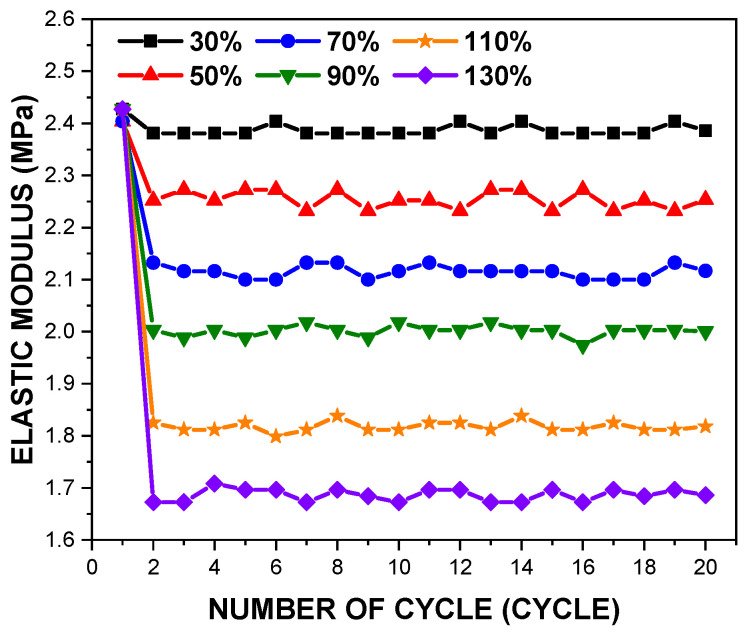
Deviations in elastic modulus of the PDMS film with a thickness of 150 μm at the elevated tensile loading cycles.

Figure 7 presents variations in elastic modulus of the PDMS film with a thickness of 150 μm with an increase in the tensile loading, and apparent differences in elastic modulus were found between the 1st cycle and the 20th cycle. The test results show a consistent monotonic elastic modulus of 2.42 ± 0.19 MPa for all specimens at the 1st cycle. Unlike a previous study [25], it was confirmed that the deviation of 50 μm in the thickness does not significantly affect the monotonic elastic modulus. In order to validate the test results, the monotonic modulus shown in Figure 6 was compared with data published by other studies.

Table 2 lists the comparisons of the monotonic elastic modulus in Figure 7 and the test results from other studies. The other studies carried out the tensile test with the same PDMS material (Sylgard 184), manufactured with the same ratio of 10:1 between the base material and curing agent. Test specimen size and the monotonic elastic modulus shown in Table 2 (a) were derived from this study and data from (b) to (d) were taken from the other studies. Two test specimens, (a) and (b), report almost the same monotonic elastic modulus, which can reliably deduce the obtained tensile test results. However, the other test specimens, (c) and (d), in bulk sizes, show less of the modulus than the specimens (a) and (b), confirming that the larger the specimen, the smaller the monotonic elastic modulus.

**Figure 8:**
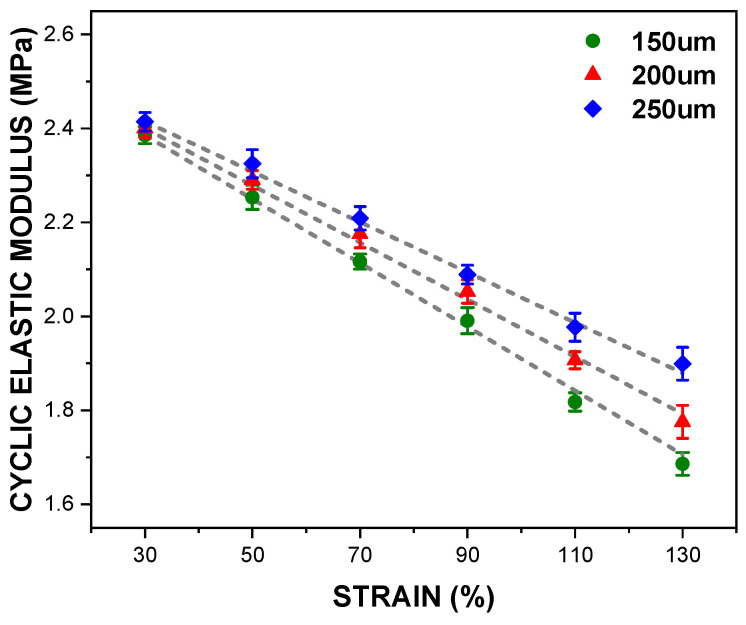
Cyclic elastic modulus against the thickness and the strain.

Figure 8 illustrates reductions in elastic modulus of the PDMS films during the loading cycles, as the magnitude of the tensile loading increases. Over the loading cycles, elastic modulus becomes stabilised with deviation of 3%, and becomes averaged as cyclic elastic modulus, as listed in Table 3. Contrasting to the deviation of the monotonic elastic modulus, cyclic elastic modulus tends to increase with an increase in film thickness. To sum up, regardless changes in the thickness and the magnitude of the tensile loading, the PDMS films have a constant monotonic elastic modulus, but cyclic elastic modulus is altered.

Therefore, when simulating PDMS-film-based products subjected to cyclic loading, the mechanical behaviours of the products should be analysed using material coefficient parameters that can mimic the cyclic elastic modulus.

## 4. Hyperelastic Material Coefficient Fitting

**Figure 9:**
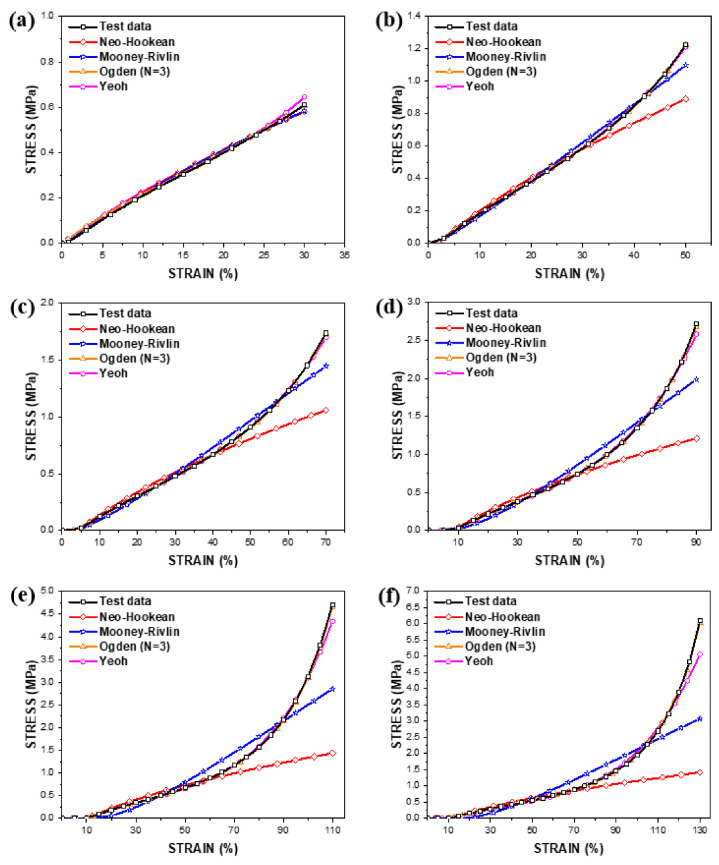
Hyperelastic curve fittings for the PDMS film with a thickness of 150 μm: (**a**) 30%, (**b**) 50%, (**c**) 70%, (**d**) 90%, (**e**) 110%, (**f**) 130%.

Figure 9 shows curve-fitting results that simulate the hyperelastic behaviours of the PDMS film with a thickness of 150 μm against an increase in the tensile loading, using four material models: the neo-Hookean model, the Mooney–Rivlin model, the third-order Ogden model and the Yeoh model. Esmail et al. (2020) presented several material hyperelastic models using Abaqus software [28]. The curve fittings were conducted based on the stress–strain test data at the 20th cycle. At a tensile loading of 30%, all four material models mimic the hyperelastic behaviour with the least difference. The neo-Hookean and Mooney–Rivlin models showed a deviation in the behaviour after the tensile loading of 50% and the difference became larger as the tensile loading increased. The Yeo model started to deviate from the curve after the tensile loading of 90%, and the error rose as the tensile loading increased. However, the third-order Ogden model simulated the hyperelastic behaviour most similarly to the test data within all tensile loading ranges.

Table 4 summarises material coefficients that simulate the hyperelastic behaviours of the PDMS films at individual tensile loading. The constants, representing the compressibility of the material, were assumed to be zero since the PDMS films were hardly compressible. The curve-fitting results demonstrated that the third-order Ogden model is preferable for the analysis of the PDMS films over the whole tensile loading range.

## 5. Conclusions

This research aimed to define the mechanical properties of the PDMS films against both monotonic and cyclic loading. The effects of parameters such as the tensile strain and the specimen thickness on the mechanical properties of the films were investigated. The experiment results are summarised as follows:

- For the tensile strain ranges between 30% and 90%, the peak stress was nearly the same during the cyclic loading, but a significant reduction was observed in tensile strain ranges greater than 90%.
- At the first cycle, the monotonic elastic modulus was almost consistent for all specimens, which confirmed that the deviation of 50 μm in the thickness did not significantly affect the monotonic elastic modulus.
- In contrast to the monotonic elastic modulus, the cyclic elastic modulus tended to escalate as the film thickness increased, but tended to decrease with an increase in the tensile loading.
- Hyperelastic material coefficients were defined to simulate the cyclic mechanical behaviours of the PDMS films. The third-order Ogden model showed the best fitting results over the entire tensile strain range.

Lastly, the experiment results present an insight into the differences in mechanical behaviours under monotonic and cyclic loadings. Moreover, designers can utilise the hyperplastic material coefficients for the structural integrity assessment of PDMS-film-based products.

## Figures and Tables

**Table 1:** Experimental parameters of the cyclic tensile test.

Parameters	Values
Standard test method	ASTM-D882
Specimen materials	PDMS (Polydimethylsiloxane)
Thickness	150, 200, 250 ± 10 (μm)
Width × Length	10 (mm) × 100 (mm)
Distance between grips	50 (mm)
Test equipment	E-3000, Illinois Tool Works Inc.
Strain increase	30–130, step 20 (%)
Strain rate	10 (mm/min)
Repeatability	5 (times)
Cycle	20 (cycles)

**Table 2:** Comparison of monotonic elastic modulus with other studies.

Specimen Dimensions (mm) (Length × Width × Thickness)			Monotonic Elastic Modulus (MPa)
(a)	100 × 10 × 0.2		2.42 ± 0.19
(b)	115 × 5 × 0.2	[15]	2.46 ± 0.16
(c)	75 × 6 × 4	[26]	1.58 ± 0.10
(d)	75 × 12.5 × 2	[27]	1.82 ± 0.10

**Table 3:** Summary of cyclic elastic modulus of the PDMS films.

Strain (%)	Cyclic Elastic Modulus (MPa)		
	Thickness of the PDMS Films (μm)		
	150	200	250
30	2.39	2.40	2.41
50	2.25	2.29	2.32
70	2.12	2.18	2.21
90	1.99	2.05	2.09
110	1.82	1.91	1.98
130	1.69	1.78	1.90

**Table 4:** Coefficient of the hyperelastic material models against tensile loadings.

Third-Order Ogden Model							
Specimen Thickness (μm)	Hyper Elastic Material Coefficient	Tensile Strain (%)					
		30	50	70	90	110	130
150	$\mu_{1}$	−5.594711	−2.348676	−1.409677	−2.384077	0.263213	0.604602
	$\alpha_{1}$	−5.182573	8.043136	6.210769	6.217385	4.907022	1.945838
	$\mu_{2}$	3.229671	0.728314	0.204002	0.328402	0.001876	0.140047
	$\alpha_{2}$	−2.907440	9.906084	9.192678	8.561867	14.191971	12.334158
	$\mu_{3}$	3.322678	2.375117	1.898027	2.695122	0.454483	−0.267031
	$\alpha_{3}$	−8.528927	6.062830	4.763705	5.250434	−5.828099	−24.725894
200	$\mu_{1}$	−0.295821	1.654135	0.590544	1.013227	3.203418	0.584670
	$\alpha_{1}$	2.001144	−13.686336	1.578236	−9.281674	−0.888450	1.789209
	$\mu_{2}$	0.654140	0.629338	0.001385	0.434336	0.029540	0.239936
	$\alpha_{2}$	4.003101	13.186393	15.362390	12.533238	10.234323	12.480701
	$\mu_{3}$	0.419904	−1.727669	0.270030	−0.873365	−2.746224	−0.466616
	$\alpha_{3}$	−1.997385	−25.120702	−14.913937	−24.999502	−2.429734	−24.999846
250	$\mu_{1}$	−3.321326	−1.979203	1.188739	1.242766	0.164472	0.450567
	$\alpha_{1}$	1.994642	4.852943	−9.402663	−8.051210	7.220017	1.108518
	$\mu_{2}$	2.074812	1.125128	0.487679	0.743862	0.000974	0.000450
	$\alpha_{2}$	3.995351	6.456453	12.542545	12.490390	15.149941	15.239930
	$\mu_{3}$	2.102843	1.652740	−0.985134	−1.457221	0.605288	0.270454
	$\alpha_{3}$	−2.005924	2.141025	−24.996848	−24.998148	−0.651284	−12.803001

## Data Availability

The data presented in this study are available on request from the corresponding author.
